# A systematic review of operational research modelling for alcohol consumption and its consequences

**DOI:** 10.1080/20476965.2025.2523751

**Published:** 2025-08-03

**Authors:** Elin H. Williams, Paul R. Harper, Geraint I. Palmer, Daniel Gartner

**Affiliations:** School of Mathematics, Cardiff University, Cardiff, UK

**Keywords:** Alcohol, literature review, Operational research

## Abstract

Recent research has revealed how operational research (OR) models and methods have been successfully applied to model alcohol consumption and its consequences (ACC). However, to date, there is no systematic review of OR methods to model ACC that can provide a broad overview of the utilisation of OR methods in this field. In this paper, we contribute to the OR literature as follows. Firstly, we provide a structured taxonomy which helps categorising the literature. Secondly, we conduct a systematic and reproducible search to identify publications that have utilised OR methods to model ACC. Thirdly, we categorise the relevant publications using the taxonomy and provide a dataset of the classification. Our findings highlight that recent research has focused on modelling consumption behaviours, particularly by utilising graph and network methods. Moreover, previous research has been predominantly led by the social sciences and public health fields and less so by the OR domain. Our results reveal gaps in the literature, including limited whole systems modelling and scarce interdisciplinary collaboration across research domains. The development of a future research agenda using our taxonomy and literature review may help closing these gaps and, ultimately, improve planning decisions to improve health, social care, and crime systems.

## 1. Introduction

Alcohol consumption and its harms within modern society is a global phenomenon (World Health Organization, 2010). Alcohol is one of the key risk factors for non-communicable diseases (Marten et al., 2020). In 2021, 1.8 million deaths were attributable to high alcohol consumption, and this was the tenth key risk factor for deaths worldwide (Institute for Health Metrics and Evaluation, 2024). Alcohol consumption was the main risk factor for medical conditions amongst 25–49 year olds in 2019 (GBD 2019 Risk Factors Collaborators, 2020). In the recent COVID-19 pandemic, alcohol consumption behaviours changed: moderate drinkers reduced their alcohol consumption whilst heavier drinkers’ consumption increased, leading to a potential surge in alcohol-related harms (University of Sheffield, 2022).

The consequences of alcohol put pressure on health systems and social care services. For example, diseases that are partially attributable and wholly attributable to alcohol are some of the health consequences of alcohol consumption (Meier et al., 2016). Violence, aggression, and criminal activity are routinely motivated by alcohol, and the relationship between alcohol and violence is well-established (Graham & Livingston, 2011), placing additional pressure on health services. Loss of productivity, absence from work, and unemployment are social consequences of alcohol consumption (Meng et al., 2014). Interactions with health, social care, and crime systems can also lead to alcohol consumption, for example, the homeless population’s difficult living conditions or the need to feel accepted within the community have a causal effect on alcohol consumption (Motta-Ochoa et al., 2023). Given the numerous consequences that result from alcohol consumption, we coin the term “alcohol consumption and its consequences”, abbreviated to ACC, which includes the numerous alcohol consumption patterns, resultant harms, and other consequences such as to the economy and taxation.

Some of the most effective interventions to impact ACC are pricing and taxation strategies (World Health Organization, 2019). The minimum unit pricing (MUP) policy is an example of a pricing strategy that sets a minimum price for alcohol per unit. The Sheffield Alcohol Policy Model (SAPM) developed at the University of Sheffield has been used to model the impacts of an MUP strategy and support its implementation in Canada, Scotland, and Wales (Brennan et al., 2015; Hill-McManus et al., 2012; Meng et al., 2014). The model comprises three stages: policy to price, price to consumption, and consumption to expected harms, depending on age, gender, consumption level, and socioeconomic group. Harms modelled include chronic/acute and wholly/partially attributable diseases, crime, and employment and work absence.

Operational research (OR), a scientific approach using mathematics and analytics to address problem-solving and decision-making problems, has been used to model various healthcare domains. Hulshof et al. (2012) provide an overview of resource capacity planning and control decisions in six healthcare services: ambulatory, emergency, surgical, inpatient, residential, and home care services. Each paper is classified by care service, planning decision level and method. Beliën et al. (2024) conducted a review of OR applied to health services over the last 50 years, discussing the main challenges and key contributions. A range of healthcare domains are reviewed – personnel scheduling, blood supply chain management, cancer diagnosis and treatment, emergency medical response and disaster relief, infectious diseases, long-term conditions, diagnostic imaging, public health, and operating room scheduling – evidencing the extent of the application of OR in healthcare settings.

Epidemiological studies of alcohol identify diseases associated with alcohol consumption including cancers (Pöschl & Seitz, 2004) and liver disease (Dang et al., 2020). ACC links to other healthcare areas, such as mental health and cancer modelling, where OR has often been applied (Alagoz et al., 2011; Brailsford et al., 2012; Noorain et al., 2019, 2022). Consequently, it is reasonable to examine the utilisation of OR methods to model ACC, one of the triggers of such health issues putting strain on public health services.

Researchers have conducted literature reviews exploring the application of OR methods in a variety of healthcare settings. The main three methods used in a sample of healthcare settings as analysed by literature reviews is presented in Table 1. Ranking the methods allows us to disregard the variety in the number of papers relevant for each literature review. Optimisation and heuristics were combined in our table as one OR area although some of the papers separated these methods.

**Table 1.** Ranked utilisation of OR for healthcare applications.

OR Method	Domain							
	Operating room planning and scheduling (Cardoen et al., 2010)	ICU management (Bai et al., 2018)	Blood supply chain (Williams et al., 2020)	Clinical pathway (Aspland et al., 2021)	Home healthcare (Grieco et al., 2021)	Frail and elderly (Williams et al., 2021)	Orthopaedic (Howells et al., 2023)	Integrated plannig in hospitals (Rachuba et al., 2024)
Decision analysis							2	
Game theory								
Graphs and networks								
Machine learning/Data mining				1				
Optimisation and heuristics	1	3	1	3	1			1
Simulation	2	1	3	2	2	2		2
Soft OR							3	
Statistical analyses						3		
Stochastic modelling		2	2		3	1	1	3

ACC differs from the other healthcare areas presented in Table 1 in that it can be a behaviour-driven issue, often requiring public health and educational interventions, rather than a process-driven problem typically addressed through scheduling, resource allocation, or optimisation interventions. Moreover, measuring outcomes in ACC contexts generally requires long-term time horizons, as many of the consequences develop or accumulate over time. Behavioural interventions also tend to necessitate extended periods of time before measurable change is observed, in contrast to scheduling and planning problems in other healthcare areas where outcomes may be more immediate or short-term.

Literature reviews exploring the utilisation of OR methods to model ACC, although there are not many, have predominantly focused on one domain, either healthcare, social care, or crime, or have not considered which domain the behaviours under consideration would affect, highlighting the need for an interdisciplinary approach (Bardach et al., 2019; Collonnaz et al., 2022; Ivaniushina & Titkova, 2021). Bardach et al. (2019) reference healthcare settings and harms, focusing on the disease burden and individual/population health associated with alcohol consumption. However, Collonnaz et al. (2022) and Ivaniushina and Titkova (2021) focus on drinking behaviours and the factors influencing consumption patterns. Collonnaz et al. (2022) excluded studies that explored health outcomes if there was no behavioural focus. OR methods could be effective in elucidating the complex relationships between these domains; however, it is unknown to what degree OR methods have been utilised to model ACC, especially in a whole systems context. Employing a whole systems modelling approach might, for example, support the identification of burdensome consumption patterns, assist researchers to better understand the complexity of the alcohol drinking system, and help identify cost-effective system-wide interventions and policies.

It is not known how OR methods have been utilised to model different populations’ consumption behaviours and the various consequences. Additionally, previous literature reviews have typically focused on the application of one, or one area of, OR methods to model ACC, obscuring the variety of methods that have, and can be utilised in this context, making the incentive and appropriateness of using such methods unclear. For example, Collonnaz et al. (2022) reviewed how network analysis methods have been utilised to model adolescents’ behaviours, focusing on one OR area and population. Similarly, Ivaniushina and Titkova (2021) explored the application of stochastic actor-based models to represent the consequences of peer influence and selection processes on adolescent alcohol consumption patterns. Nevertheless, Bardach et al. (2019) explored the utilisation of mathematical models to establish the disease burden of alcohol consumption and to conduct cost-effectiveness analyses, and the relevant literature was classified into three families of mathematical models – Markov, life-table based, and attributable fraction-based models.

Given the gaps identified in previous reviews, we conduct a literature review and provide a taxonomy to explore the application of OR methods to model ACC, to shed light on this knowledge gap. Our systematic literature review contributes to existing literature as follows:

- We provide a novel taxonomy based on various categories including descriptive statistics, modelling domain, and methodology, which supports practitioners and academics to classify the literature in this field.
- We utilise existing methods to develop a search methodology to gather relevant publications by constructing a search string incorporating alcohol drinking behaviours, consequent harms, and OR methods, and conduct a search of the Scopus database.
- Using the results of the search and the constructed taxonomy, we categorise the publications and perform full-text analyses, which support to guide a future research agenda.

The descriptive analysis reveals trends in publishing in this domain of research. Our detailed analysis helps to clarify the appropriateness of the use of OR methods in this research area, based on categories including the population modelled, the research scope, and the alcohol problem modelled, supporting practitioners and academics to recognise the advantages and disadvantages of using such methods to model ACC. By applying the taxonomy to the literature, we identify the extent of the use of interdisciplinary approaches to model ACC. We compare the classification of the relevant publications across categories of the taxonomy to determine similarities and contradictions. Additionally, the analysis supports the identification of gaps in the research field, providing motivation for future research.

We structure the remainder of our systematic literature review as follows: Section 2 presents the taxonomy that the relevant literature will be categorised by. Section 3 describes the literature review methodology including the search string constructed. In Section 4, the taxonomy is applied to the relevant literature, and the results of the full-text analyses are presented. The findings are discussed in Section 5, and the final section outlines the conclusions and areas for future research.

## 2. Taxonomy

The taxonomy described in this section is a method to classify literature based on a variety of categories, including descriptive statistics and modelling domain. Throughout this description, attribute names will be indicated in **bold** whereas attribute values will be indicated in *italics*. All attribute names, values, and definitions are summarised in Table 2. This taxonomy was assembled because a framework was needed to classify the literature applying OR methods to model ACC as to the best of our knowledge, no extensive literature review has previously been undertaken in this field.

**Table 2.** Taxonomy attribute names, values, and definitions.

Attribute Name	Attribute Values	Definition
Metadata	Year of publication Continent JCR category	2.1
Data/Information source	Primary Secondary Expert Opinion	2.2
Population	Primary/Elementary school students Adolescents University students Adults Younger adults Older adults Heavy drinkers People with alcohol use disorders Policy makers	2.3
Modelling domain	Healthcare Social care Criminal justice Other	2.4
Care area	Primary Secondary Tertiary Community	2.5
Functional area	Cost-effectiveness analysis Finance, policy/intervention, governance, regulation/funding Individual behaviours/characteristics Medical decisions Planning, system/resource utilisation Public health, community service planning Quality management, performance monitoring or review Relationship influences/modelling Workforce/staff management Other	2.6
Implementation level	Suggested/Theoretical Conceptualised Implemented	2.7
Planning decision level	Strategic Tactical Operational	2.8
Modelling scope	International National Regional	2.9
Modelling perspective	Patient Provider Societal	2.10
Modelling aims	Data collection Description Diagnosis Prognosis Optimisation	2.11
Modelling outcome	Cost Health Time Behaviours	2.12
OR method	Graphs and networks Soft OR Game theory Simulation Optimisation and heuristics Stochastic modelling Statistical analyses	2.13
Alcohol problem modelled	Consumption Consequences	2.14

### 2.1. Descriptive statistics

The descriptive statistics depict the general context and bibliographic data of publications. Three of these categories are:

#### Metadata - year of publication, continent, Clarivate Journal Citation Report (JCR) category

Definition 2.1. (Metadata) The metadata includes the publication year, the continent of the research, and the Clarivate Journal Citation Report (JCR) category in which the publication belongs to. The literature search was confined to nine JCR categories, chosen based on the objective of gathering literature applying OR methods to model ACC in healthcare, social care, and crime settings. An analysis of the JCR category supports an understanding of the research disciplines involved.

The JCR categories included in this search were:

- *Criminology & Penology (CP)*
- *Health Care Sciences & Services (HCSS)*
- *Health Policy & Services (HPS)*
- *Industrial Engineering (IE)*
- *Medical Informatics (MI)*
- *Operations Research & Management Sciences (OR/MS)*
- *Public, Environmental & Occupational Health (PEOH)*
- *Social Work (SW)*
- *Substance Abuse (SA)*

#### Data/information source

Definition 2.2. (Data/Information Source) The data or information source, classified into three categories, identifies how authors obtained the data used in their models. Data could be obtained from more than one source.

- *Primary* - Data that was collected by the authors themselves to be used in the research under consideration, for example, by conducting questionnaires, online surveys, or focus groups.
- *Secondary* - Data that was not collected by the authors themselves, but rather was collected by another source, including publicly available datasets, results acquired by other authors in published papers, and census data. This also includes routine data collection.
- *Expert opinion* - Views offered by experts in the field. This includes quantitative information such as model parameter estimations, and qualitative information, for example, advice for policy guidelines.

#### Population

Definition 2.3. (Population) The population modelled is included in the taxonomy because different factors influence the alcohol consumption, misuse, and harms experienced by different age groups and types of drinkers. The population labels were adapted from McGill et al. (2021) categorisation, and additional categories were included, namely, “adults” and “primary/elementary school students”. For details, see Appendix C. More than one population could be modelled in a paper.

### 2.2. Modelling domain

Definition 2.4. (Modelling Domain) The domain considers the setting in which research has been conducted. This category allows for investigations into the use of whole systems modelling. NHS Digital (2022) break down the healthcare domain into four care areas, whereas Brailsford et al. (2009) consider the healthcare functions of research, therefore we can further break down the modelling domain into care and functional areas.

- *Healthcare* - Modelling the progression of diseases and conditions, and the associated costs, symptoms, quality of life, and life expectancy. Healthcare also incorporates clinical trials and healthcare management.
- *Social Care* - Interpreting social care is difficult as no global definition exists (Kelly et al., 2023). It is an ever changing and evolving concept, adapting to current social issues and each region/country’s needs (Rode, 2017). Nevertheless, social care is known to encompass a variety of services including housing, mental health, and support for children. All of which assist day to day living. For the purpose of this paper, social care was defined to include social and home impacts and support such as effects on children, ACEs, maternal alcohol use, and parental support, mental health including anxiety and post-traumatic stress disorder, domestic violence, social services, or if the paper clearly specified social care.
- *Criminal Justice* - Includes police, court, prison, and probation services. Violent, aggressive, and delinquent behaviours are also involved.
- *Other* - Papers that do not explicitly model a domain, but rather model a behaviour or relationship, for example, behaviour trajectories and peer/familial influences on behaviours.

#### Care area

Definition 2.5 (Care Area) The healthcare domain can be further grouped into the care area. The care area can be described by the care provider to provide insights into the organisation providing services, which can be grouped as described by NHS Digital (2022):

- *Primary* - Includes general practice and dental services.
- *Secondary* - Both elective and emergency care. This also includes mental health care.
- *Tertiary* - This is specialised treatment to treat a specific complex health condition, including organ transplants and dialysis.
- *Community* - These are services provided in the community, such as district nursing and child health services.

#### Functional area

Definition 2.6 (Functional Area) The functional area, based on the categorisation of healthcare functions described by Brailsford et al. (2009), denotes the purpose of the research within a healthcare, social care, or crime domain. Additional categories were added to the categorisation proposed by Brailsford et al. (2009) to incorporate functions pertinent to ACC. The categories “Cost-effectiveness analysis”, “Medical decisions”, and “Relationship influences/modelling” were additional:

- *Cost-effectiveness analysis*
- *Finance, policy/intervention, governance, regulation/funding*
- *Individual behaviours/characteristics*
- *Medical decisions*
- *Planning, system/resource utilisation*
- *Public health, community service planning*
- *Quality management, performance monitoring or review*
- *Relationship influences/modelling*
- *Workforce/staff management*
- *Other*

### 2.3. Implementation level

Definition 2.7. (Implementation Level) The implementation level definitions developed by Brailsford et al. (2009) and adapted by Howells et al. (2023) have been used in this analysis. This category infers a relationship between the research findings and their application in practice. The implementation level of models developed and utilised can be categorised into three classes:

- *Suggested/Theoretical* - Theoretically proposed by the authors, with no indication of the level of implementation of the model.
- *Conceptualised* - Discussed with a client or organisation, or based on an established model, however, has not yet been implemented in practice.
- *Implemented* - A model which has been implemented in practice or analysing a system that has already been implemented.

### 2.4. Planning decision level

Definition 2.8. (Planning Decision Level) A taxonomy to classify planning and control decisions within healthcare delivery organisation is presented by Hulshof et al. (2012). The planning decision level can be classified into one, or more than one, of three temporal levels – strategic, tactical and operational. The operational level is broken down into two sub-categories, namely online and offline, referring to when decisions are made.

- *Strategic* - Decisions based on long planning horizons. These are structural decisions, including defining an organisation’s mission. Information utilised to make these decisions is based on forecasts and aggregated data. An example of a strategic problem is facility location planning.
- *Tactical* - Decisions based on medium-term planning horizons. This planning level involves translating decisions made at the strategic level into guidelines. Tactical problems comprise of organising the implementation of delivery processes. The information on which these decisions are made is partly forecasted. Some tactical problems are staff-shift and surgical block scheduling.
- *Operational* - Short-term decision-making. Decisions are made on the individual patient or resource level. The information on which these decisions are made is known. Operational planning can be classified further:
  - *Online* - Decisions made to react to unplanned events, for example, rescheduling elective patients to accommodate for emergency patients.
  - *Offline* - Advance planning decisions based on current elective demand, for example, assigning patients to appointments.

### 2.5. Modelling and methodology

#### Modelling scope

Definition 2.9. (Modelling Scope) The modelling scope is based on geographical coverage of the sample utilised in the model, and refers to the generalisability of the results obtained. The scope is also an indication of the geographical variability of ACC.

- *International* - Research constituting of more than one country, or a globally representative sample.
- *National* - Studies utilising participants from, or samples representative of, a whole country.
- *Regional* - An area smaller than national, including schools, towns, and cities.

Modelling perspective

Definition 2.10. (Modelling Perspective) The modelling perspective identifies the viewpoint in which the research is being considered from. Research can be conducted from more than one perspective.

- *Patient* - Modelling a cohort of patients, for example, a group of patients diagnosed with a condition. This also incorporates patient disease progression and analyses of financial costs for patients.
- *Provider* - Modelled from the perspective of an organisation providing a service. This includes modelling healthcare, social care, and crime staff, services, and utilisation.
- *Societal* - Modelling from a wider, more general perspective, including population studies. Societal perspective can also include loss of productivity and work absenteeism.

#### Modelling aims

**Figure 1.**
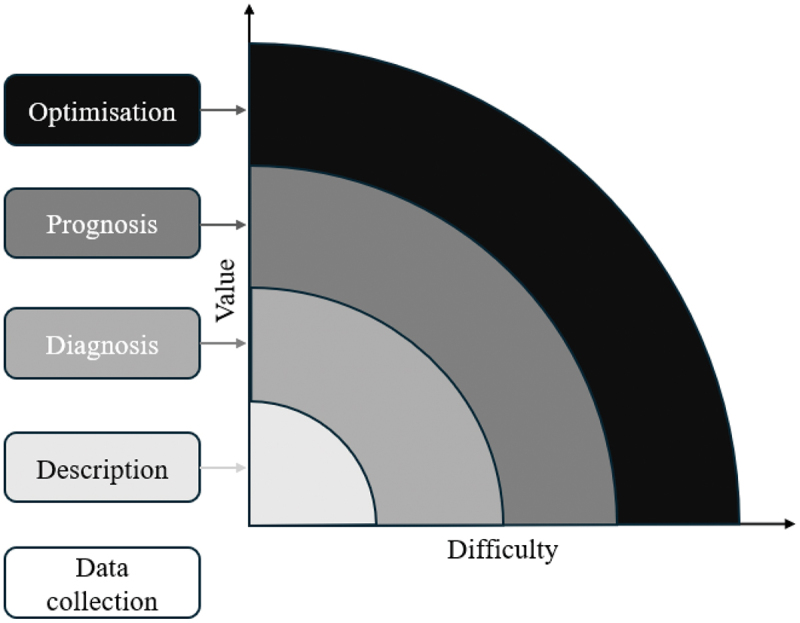
Levels of modelling aims.

Definition 2.11. (Modelling Aims) A publication’s objective can be summarised by the modelling aim, shown in Figure 1. This has been adapted from business value complexity and level of analytics classifications that is typically used in supply chain risk management. The labels and definitions are based on a variation of the categorisation described by Simchi-Levi (2015), which have been modified to align with the focus of this literature review.

This description’s labels are defined as follows:

- *Data collection* - What data? Collect, clean, format, and structure
- *Description* - What has happened? Reports, alerts, mapping
- *Diagnosis* - Why did this happen? Queries, data mining, statistical analysis
- *Prognosis* - What will happen? Forecasts, machine learning, simulation
- *Optimisation* - How do we get there? Optimisation, planning

#### Modelling outcome

Definition 2.12. (Modelling Outcome) The desired or acquired outputs of a model can be categorised into four classes and help us to identify the greatest concerns within ACC:

- *Cost* - Financial costs, including costs to individuals or associations. This includes cost-effectiveness analyses and modelling cost implications of policies and interventions.
- *Health* - The outcome of interest is individuals’ health. Studies could be modelling disease progression or medication effectiveness. QALYs are also considered as a health outcome.
- *Time* - The outcome of interest relates to time, for example, time until an event such as readmission. Time outcomes also include staff and patient scheduling and waiting times.
- *Behaviours* - The publication’s outcome concerns the adoption, change, or continuation of behaviours. Papers may consider behaviour trajectories, how peer groups can encourage or discourage behaviours, or investigations of individual behaviours following interventions.

#### OR method

Definition 2.13. (OR Method) Categorising the OR method used provides insights into the utilisation of OR methods in the field. When more than one method has been utilised, this category is separated into two sub-categories – primary and secondary OR method. The methods are based on previous literature reviews in the application of OR to model healthcare settings (Cardoen et al., 2010; Grieco et al., 2021; Howells et al., 2023; Noorain et al., 2022; Palmer et al., 2017; Williams et al., 2021) and are grouped into OR areas. The OR areas and some methods belonging to each area are as follows:

- *Graphs and networks* - graph theory, stochastic actor-based models, network analysis
- *Soft OR* - Delphi method, Strategic Options Development and Analysis (SODA)
- *Game theory*
- *Simulation* - agent-based model, system dynamics, Monte Carlo, microsimulation, discrete event simulation
- *Optimisation and heuristics* - mathematical programming, metaheuristics, linear programming, Tabu search, branch, and bound
- *Stochastic modelling* - Markov methods, queueing theory
- *Statistical analyses* - used when a primary statistical method is utilised in combination with an OR method as a secondary method

### 2.6. Alcohol problem modelled

Definition 2.14. (Alcohol Problem Modelled) The alcohol-related issue modelled is broadly categorised as the consumption or the consequences of drinking alcohol. It is important to consider the problem modelled to provide a distinction between consumption and consequences, as these can vary depending on contextual factors, some of which are included in this taxonomy.

- *Consumption* - This comprises modelling alcohol consumption behaviours and patterns, including binge drinking, hazardous and harmful consumption, family and peer influences on consumption, alcohol use disorder (characterised by a difficulty in controlling alcohol consumption (National Institute on Alcohol Abuse and Alcoholism, 2020)), and intervention or policy modelling to alter consumption behaviours.
- *Consequences* - This category consists of studies investigating the consequent health, social care, and crime burdens of drinking alcohol. Consequences include partially and wholly attributable alcohol diseases, alcohol-related crime, violence and aggression, and social harms such as homelessness and work absenteeism.

## 3. Search methodology

The methodology presented in this section describes the process of searching for relevant publications and screening the texts to identify those applicable for a full-text analysis. A search string was constructed in Scopus, a search engine and abstract and citation database, to gather literature modelling ACC using OR methods. The search string consisted of two term categories: one category of alcohol drinking behaviours and consequent harms and one category of OR methods (the full list of terms used is available in Appendix A, Table A1). The alcohol-related terms were drawn from a range of literature reviews (Kõlves et al., 2020; Murray et al., 2022; Newbury-Birch et al., 2009; Probst, et al., 2020). We did not include “alcohol” in the search string because this was considered to be too broad. The OR methods were selected by incorporating terms used in previous literature reviews in the application of OR methods to model healthcare settings (Cardoen et al., 2010; Grieco et al., 2021; Howells et al., 2023; Palmer et al., 2017; Williams et al., 2021). We did not directly take the OR terms used in previous literature reviews in the application of OR methods to model ACC because each paper was focused on one, or one area of, OR methods (Bardach et al., 2019; Collonnaz et al., 2022; Ivaniushina & Titkova, 2021).

The search was confined to nine Clarivate JCR categories to filter out as much irrelevant literature as possible. The journal categories were selected based on the objective to identify literature applying OR methods to model ACC in healthcare, social care, and/or crime domains. No restriction was placed on the literature’s publication year.

The search string was constructed based on the Scopus Search Guide (Elsevier). The asterisk (*) symbol was used in the search string to allow terms to have alternative endings, for example, “binge drink*” could return “binge drinker”, “binge drinking”, or any other combination of letters following “drink”. The dollar ($) symbol was used to allow the final letter of a word to vary, for example, “metaheuristic$” could return “metaheuristic” or “metaheuristics”.

Screening was based on the literature’s titles, abstracts, and key words. For literature to be identified as relevant, at least one OR method and one alcohol drinking behaviour or alcohol-related harm must have been mentioned in the title, abstract, or key words. Only studies examining human behaviours were considered relevant – biochemical research and animal studies were excluded. Literature that did not differentiate between alcohol and other substances, for example opioids and tobacco, were excluded because of uncertainty regarding the generalisability of results. Other substances differ to alcohol because of their legal status and cultural use (Dwight, 2001).

Initially, 960 documents were identified from the Scopus database search, resulting from the constructed search string. The original search was conducted on the 10th of January 2024. Following screening the title, abstracts and key words of the original 960 documents, 219 documents were identified as relevant. The full-text of the relevant literature was screened to select the literature applicable for full-text analysis. The search was further reduced to 105 publications being applicable for full-text analysis. This included five literature review papers, which were not included in the analysis, but were used in the forward and backward searches. Therefore, a total of 100 papers from the original search were included in the analysis. Articles often required clarification of which substances were considered, or whether results were presented separately for the range of substances investigated, explaining the difference in the number of articles identified as relevant and those selected for full-text analysis. During the full-text reading, the fields of the taxonomy were completed in an .csv file, which could later be used for analysis.

**Figure 2.**
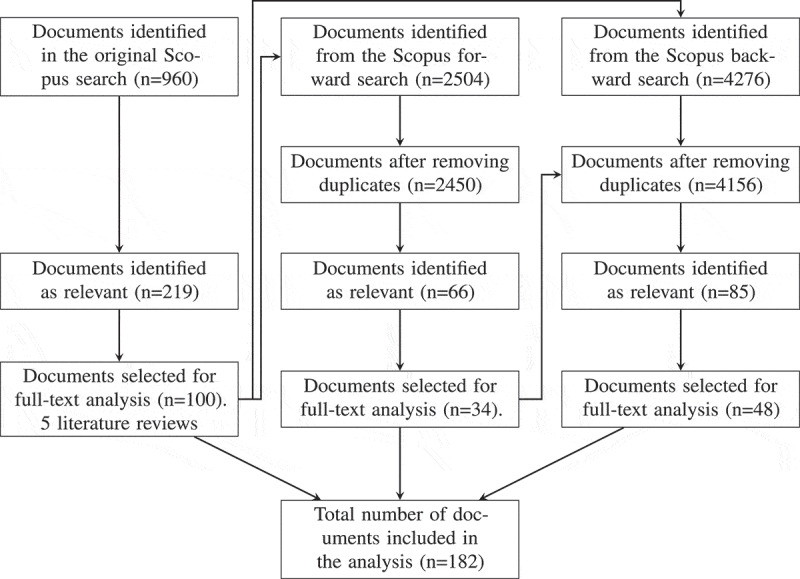
Flow chart of the search and screening methodology.

Forward and backward searches were conducted on the 28th of February 2024 on the 100 publications selected for full-text analysis and the five relevant literature review papers. A forward search was conducted to gather literature which had cited these 105 papers (Webster & Watson, 2002). In total, 2504 publications were identified in the forward search, with 54 duplicates from the original Scopus search, resulting in 2450 new publications. Following screening the titles, abstracts and key words of publications from the forward search, 66 documents were identified as relevant and of these, 34 were included in the full-text analysis. Finally, a backward search was conducted to explore the citations of the original 105 publications (Webster & Watson, 2002). In total, 4276 documents were identified in the backward search, with a total of 120 duplicates across the original and forward searches, resulting in a total of 4156 new articles to screen. After screening the titles, abstracts and key words of the publications in the backward search, 85 were identified as relevant and of these, 48 were selected for full-text analysis. The search and screening process is illustrated in Figure 2.

The search and screening process resulted in 182 publications being relevant for full-text analysis. These final 182 articles were taxonomised based on the taxonomy presented in Section 2

## 4. Findings

This section presents the findings of the analysis of the relevant literature from the original, forward and backward searches, and follows the taxonomy constructed in Section 2

### 4.1. Descriptive statistics

**Figure 3.**
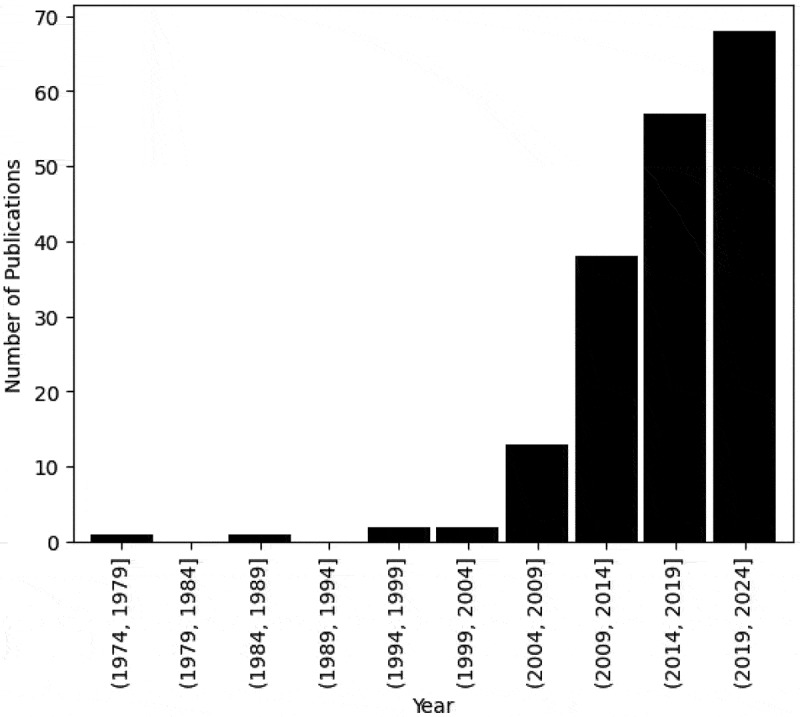
Number of publications by publication year.

The publication date of the relevant literature ranges from 1979 to 2024 (Figure 3), remembering that at the time of the analysis the year 2024 was not complete. From 2005 to 2009, there has been a continuous increase in the number of publications utilising OR methods to model ACC.

**Figure 4.**
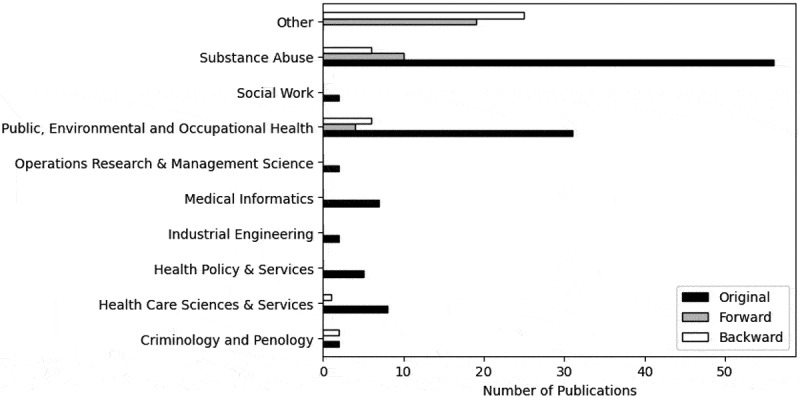
Number of publications by JCR category and search.

The literature is classified by JCR category and is presented by the original, forward and backward searches. The majority of publications from the original search are published under the “Substance Abuse” and “Public, Environmental and Occupation Health” categories, whereas a significant proportion of publications from the forward and backward searches are under “Other” JCR categories, shown in Figure 4. The “Substance Abuse” category consists of 49.00%, 30.30%, and 15.00% of publications from the original, forward and backward searches, respectively. Furthermore, no publications from the forward or backward searches were published under the “Health Policy & Services”, “Industrial Engineering”, “Medical Informatics”, “Operations Research & Management Science”, or “Social Work” categories. However, 57.58% and 62.50% of the literature from the forward and backward searches, respectively, belonged to JCR categories not incorporated into the search string.

**Figure 5.**
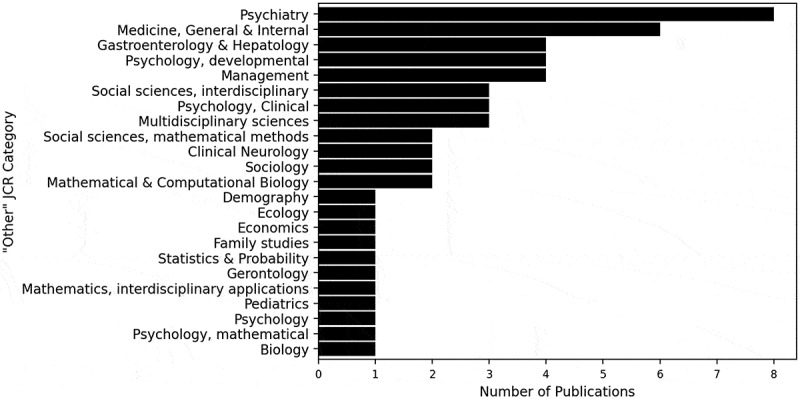
Number of publications in ‘Other’ JCR categories.

A more detailed analysis of the JCR classification discloses that the publications from “Other” journals belong under a wide variety of JCR categories, with a maximum number of eight publications belonging to any one JCR category, shown in Figure 5. The publications belong to an additional 23 JCR categories, where “Psychiatry” is the “Other” JCR category with the most publications. The research involves many different research disciplines including “Biology”, “Economics”, and “Psychology”, depicting the breadth of this area of study and its cross-disciplinary applications (the full list of journals is provided in Appendix B, Table B1).

**Figure 6.**
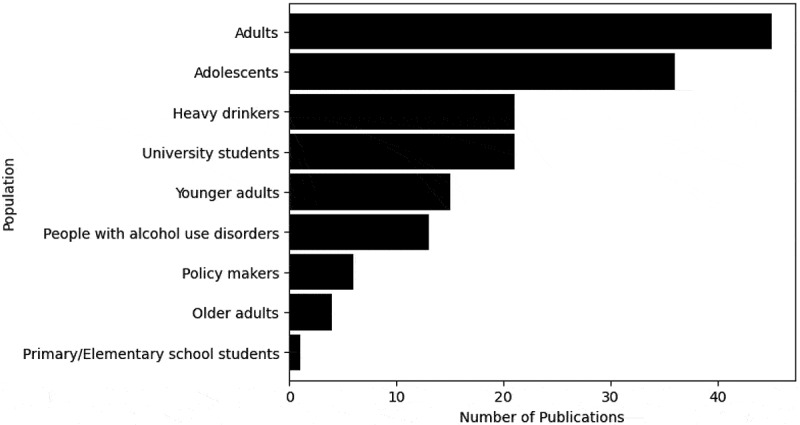
Number of publications by the population modelled.

The population modelled is classified by age categories, groups of drinkers and policy makers. As shown in Figure 6, the adult population is the most commonly modelled, indicating that publications are frequently based on general populations. For example, Gibbs et al. (2022) modelled the effects of a minimum unit pricing strategy on harms on the adult population, 15 years and older, of South Africa, including alcohol consumption and expenditure, mortality and workplace absenteeism, based on poorness and richness. Adolescent populations are also commonly modelled, evidencing how critical adolescence can be in shaping individuals’ future, including future consequences of their alcohol consumption. Some studies focus on groups of drinkers, for example, Julien et al. (2020) projected the prevalence of alcohol-related liver disease in a population of heavy drinkers.

Most publications utilised secondary data sources (69.35% of papers) to populate the studies and models. Publications utilising primary data sources (22.58% of papers) often involved the construction of networks (Balestrieri et al., 2018; D’Amico et al., 2023; Karnick et al., 2022; Meisel et al., 2018), whereas the publications utilising expert opinion (8.06% of papers) included systems mapping (Hosseinichimeh et al., 2022; Matson et al., 2022; Salmon et al., 2020) and developing guidelines (Kingston et al., 2009).

### 4.2. Modelling domain

**Figure 7.**
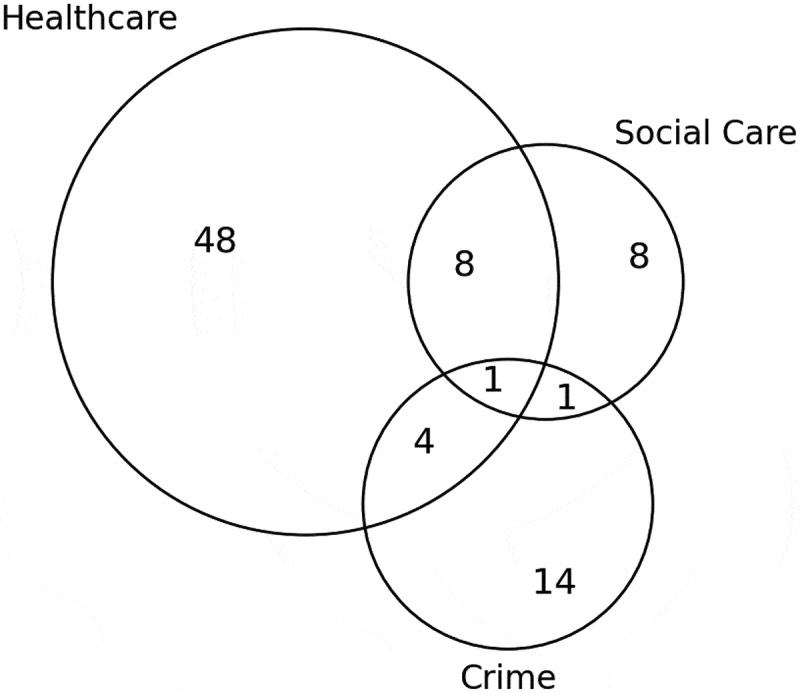
Number of publications by the modelling domain.

The modelling domain includes healthcare, social care, and crime services. Figure 7 shows that healthcare settings in isolation are the most commonly modelled domain.

Substantially less literature models social care and crime domains. Only nine and six publications model social care and crime domains, respectively, in relation to any other setting. Only one publication considers all three domains – Holder’s (1998) publication portrays the system of alcohol consumption, its demand on social and health services, and its consequences on criminal harms such as drink driving. Those papers conducted within healthcare domains considered the effects of alcohol consumption on a range of health conditions and diseases including HIV (Lesko et al., 2023; Wong et al., 2013) and alcohol-related liver disease (Julien et al., 2020; Wu et al., 2023). Furthermore, various measures of the success of healthcare treatments such as QALYs (Barbosa et al., 2010) and cost-effectiveness (Barbosa et al., 2023; Laramee et al., 2014; Laramée et al., 2016) were considered. Papers considering crime domains typically examined drink-driving (de Carvalho Ponce et al., 2018; Hosseinichimeh et al., 2022; Salmon et al., 2020; Summers & Harris, 1979) or violent/aggressive behaviours (Cerdá et al., 2022; Redfern et al., 2017; Scott et al., 2017; Scott et al., 2016). Mental health conditions including PTSD (Afzali et al., 2017; Li et al., 2024; McGlinchey et al., 2022; Sistad et al., 2023), ACEs and other childhood impacts (González-Alcaide et al., 2016; White et al., 2008; Zhao et al., 2023), and social services (Wilson et al., 2016) were noted under social care.

The healthcare domain can be further divided into the care area. Publications were modelled across a range of care settings where some papers considered more than one care area. Secondary care was the most frequently modelled area with 38.46% of papers, followed by community care (26.92%), primary care (23.08%), and lastly tertiary care (11.54%). Secondary care modelling included emergency department settings (Bernstein et al., 2023; Wilson et al., 2016) and outpatient clinics (Poon et al., 2022). Nevertheless, only a relatively small number of papers specifically describe the care area modelled. Twenty-three papers describe the care area, where some model more than one care area, which only accounts for 47.92% of the papers modelling healthcare domains.

**Figure 8.**
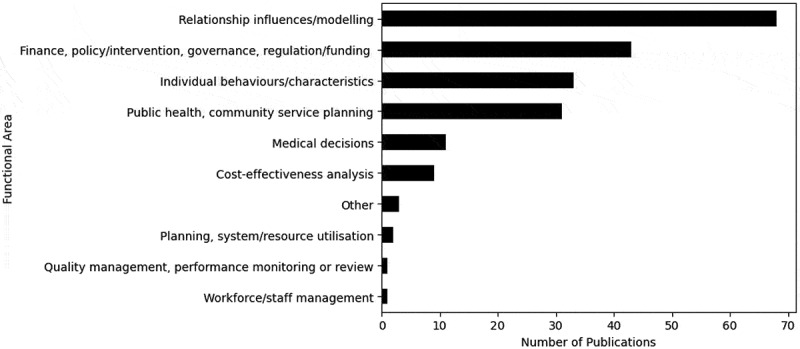
Number of publications by the functional area.

In our taxonomy in Section 2, we included the functional area as part of the modelling domain to denote the purpose of the research within a healthcare, social care, or crime domain. The most common functional area is relationship influences/modelling, shown in Figure 8, where the research often looks at alcohol’s influence on adolescent friendship formations, and friends’ influence on alcohol consumption (Hoeben et al., 2021; Light et al., 2019; Long et al., 2017). Numerous publications’ functional area is finance, policy/intervention, governance, or regulation/funding, where these papers frequently model the effectiveness of alcohol taxation policies on alcohol consumption and the related harms. For example, Keyes et al. (2019) approximated the impact of alcohol taxation on violence and homicide. Only a small number of publications consider workforce or staff management (McEachern et al., 2016), quality management (Conlin et al., 2022), or planning or system and resource utilisation (Kools et al., 2022).

### 4.3. Implementation level

**Figure 9.**
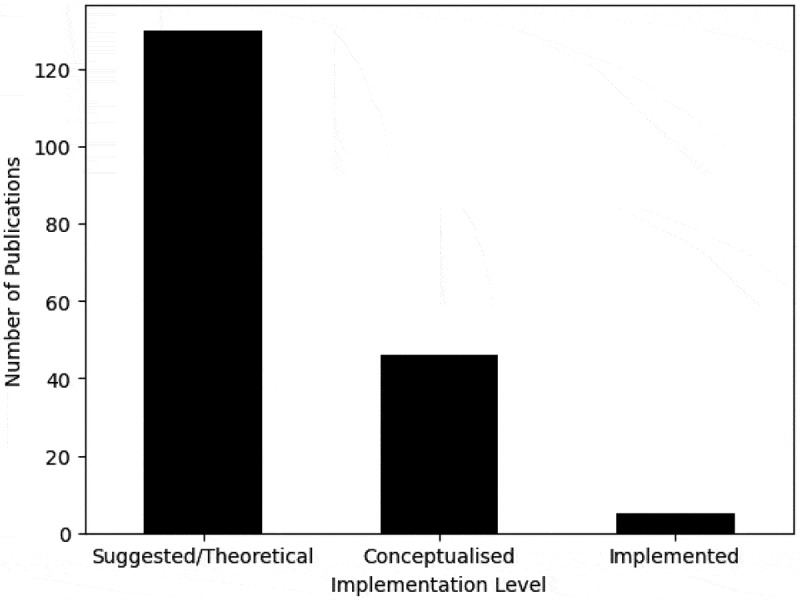
Number of publications by the implementation level.

Figure 9 illustrates the implementation level of the literature, classified as theoretical, conceptualised, or implemented.

The majority of publications are theoretical, comprising 71.82% of publications, 25.41% are conceptualised, however, only 2.76% have actually been implemented. Unfortunately, this is a trend commonly seen in studies on the application of OR methods to model healthcare settings (Howells et al., 2023; Hulshof et al., 2012). Those classed as “implemented” evaluated systems and policies already implemented, for example, Kools et al. (2022) evaluated an interdisciplinary AUD treatment team that had been implemented in a hospital, and Hilton et al. (2020) and Fergie et al. (2019) modelled the discourse around an implemented MUP policy.

### 4.4. Planning decision level

With respect to the 31 publications that consider planning and control decisions, the majority assess strategic decisions (64.52% of the papers applicable), 10 consider tactical decisions (32.26% of the papers), and only one examines operational planning (3.23% of the papers), where this is an offline decision. Many of the interventions tested in these 31 publications are long-term taxation and pricing policies, including the strategies modelled by Korhonen & Soismaam (1988), Stankov et al. (2019), and Stacey et al. (2018), each strategy having the goal of reducing long-term alcohol-related harms.

### 4.5. Modelling and methodology

The scope denotes the breadth of the research and the extent to which the problem at hand has been studied. Most publications model at a regional scope, followed by national and then international.

Healthcare is often modelled at a national scope, as illustrated in Table 3, which might be an indication of how healthcare services are usually organised. For example, Julien et al. (2020) projected the number of alcohol-related liver cirrhosis deaths across the United States. Contrarily, crime is predominantly modelled at a regional scope. The “other” setting, often encompassing modelling alcohol consumption behaviours, is regularly undertaken on a regional scope.

**Table 3.** Number of publications by modelling scope and domain.

Scope	Domain				Total
	Healthcare	Social Care	Crime	Other	
International	5	2	0	5	12
National	28	6	2	24	60
Regional	17	8	13	53	91
Total	50	16	15	82	163

The majority of publications model from a societal perspective (71.36% of papers), indicating alcohol’s significant presence in communities and its substantial burden on society. Publications that model from a societal perspective consider societal harms such as work absenteeism (Gibbs et al., 2022) and loss of productivity (Julien et al., 2024), in addition to alcohol’s presence within communities and networks (Hallgren et al., 2017; Sánchez et al., 2007; van den Ende et al., 2024). Significantly less publications model from the patient (14.56% of papers) or provider’s (14.08% of papers) perspective, with 14.56% and 14.08% of publications modelling from these perspectives, respectively.

**Figure 10.**
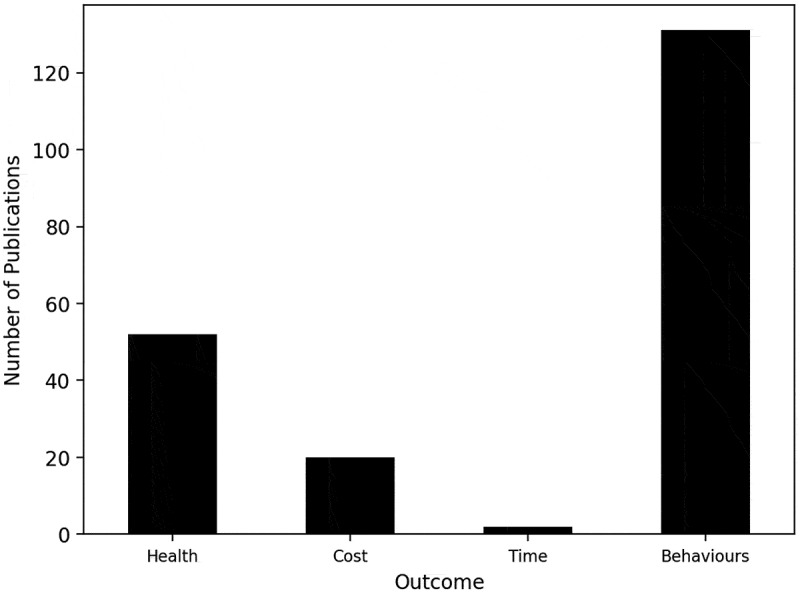
Bar chart of the number of publications by the modelling outcome.

The outcome of the majority of publications is behaviours, including individual drinking behaviour trajectories (Fortin et al., 2016; Witkiewitz et al., 2014) and alcohol consumption behaviours within social networks (Balestrieri et al., 2018; Mundt et al., 2012; van den Ende et al., 2024; Wang et al., 2016) (Figure 10). Many publications model health outcomes, for example, Julien et al. (2020) project the prevalence of alcoholic-related cirrhosis and consequent deaths, and predict a 75% increase in alcohol-related liver disease deaths by 2040 without any change to current alcohol consumption behaviours. Cost outcomes are not frequently examined, whilst outcomes relating to time are rarely studied.

**Figure 11.**
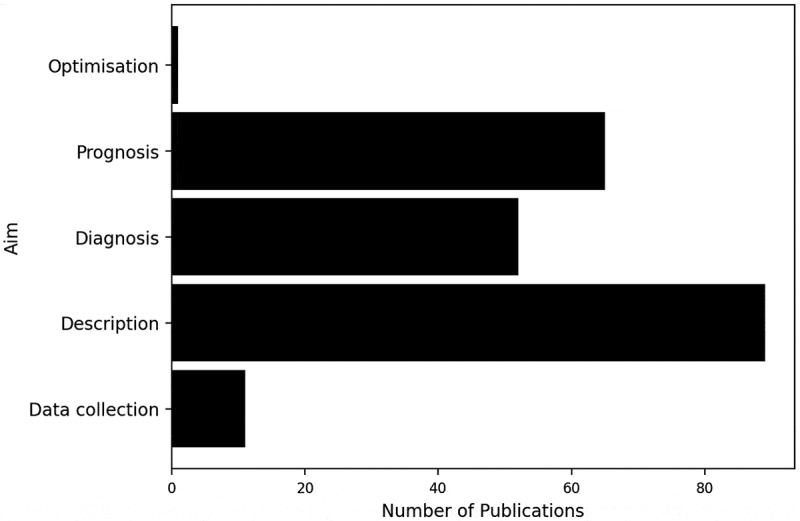
Bar chart of the number of publications by the modelling aim.

Figure 11 shows that the aim of the publications is most frequently description, followed by prognosis and diagnosis. Those publications with a descriptive aim commonly describe the prevalence of alcohol consumption within social and friendship networks (Long et al., 2017; Meisel et al., 2018; Ragan, 2020; Van Ryzin et al., 2016). Only one publication’s aim is optimisation – this is a paper by Korhonen and Soismaam (1988) who develop a multi-objective program to determine the optimal price for alcohol.

The methodology is categorised by various OR areas, and each OR area can be broken down into the specific OR method utilised, outlined in Table 4 (the full list of publications by the OR area and method used is provided in Appendix D, Table D1). Graph and network methods are the most utilised OR area to model ACC, highlighting the substantial influence of social and peer networks on alcohol consumption and related harms. Simulation methods are also frequently used, including agent-based modelling, system dynamics, and Monte Carlo simulation, as shown in Table 4. Stochastic modelling is repeatedly used to model the effectiveness of medications and treatments on alcohol consumption and craving (Berman et al., 2019; Book et al., 2005; Laramée et al., 2016; Prisciandaro et al., 2011; Prisciandaro et al., 2012; Shirley et al., 2010; Sluiter et al., 2018; Witkiewitz et al., 2010).

**Table 4.** Number of publications by the OR area and method used.

OR Area	N	Method	n
Graphs and Networks	66	Network analysis Stochastic actor-based/oriented model	46 20
Simulation	50	Agent-based model System dynamics Microsimulation Monte Carlo simulation Other	27 11 6 2 5
Stochastic modelling	37	Markov model Markov chain Markov process	34 2 1
Statistical methods	22		22
Soft OR	4	Delphi method	4
Game theory	3		3
Optimisation and Heuristics	1	Goal program/Multi-objective program	1

A range of OR areas have also been utilised as a secondary method (Table 5), especially when statistical methods are the primary method. Simulation methods are the most recurrently used secondary OR area, where the method utilised is often Monte Carlo simulation (Byrnes et al., 2010; Joneydi et al., 2023; Shield et al., 2020). Monte Carlo simulation is commonly used to measure the uncertainty of models. Two of the papers utilising optimisation and heuristics as a secondary method used a simulated annealing algorithm, and the other paper employed a multi-objective program, each to calculate the parameters and calibrate the primary model. Stochastic modelling methods were interpolated in the primary models to inform transition probabilities.

**Table 5.** Number of publications by the secondary method.

Secondary Method	Graphs and Networks	Optimisation and Heuristics	Simulation	Stochastic Modelling
Number of Publications	1	3	29	2

**Figure 12.**
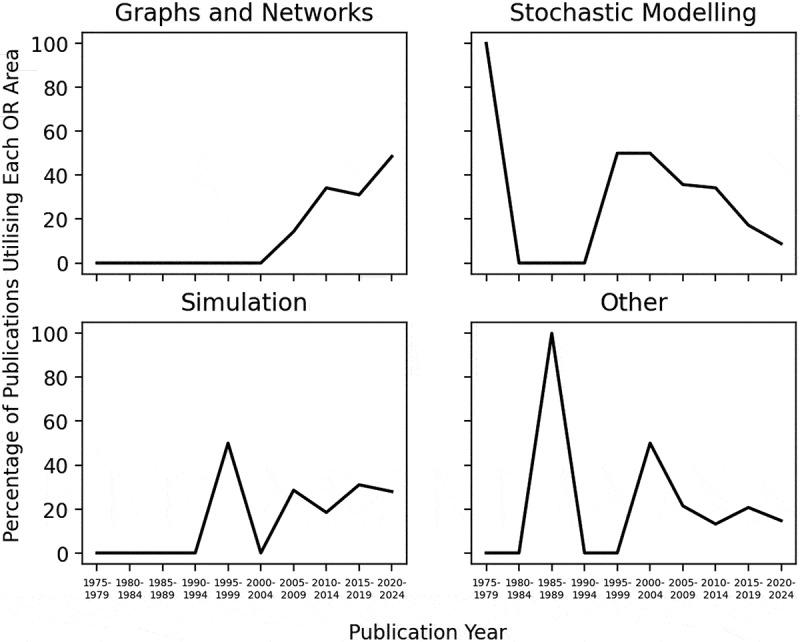
Percentage of publications utilising each OR area by publication year.

Figure 12 illustrates the percentage of publications utilising each OR area by publication year. The use of graph and network methods has steadily increased since 2005–2009 and is the dominating method by 2020–2024 with 48.53% of publications from these years utilising graph and network methods, suggesting a growing interest in network-based knowledge of ACC. The utilisation of stochastic modelling methods has experienced the opposite trend to that of graph and network methods, with its most dominant use in 1995–1999 and decreasing since. Simulation methods saw its most dominant use in 1995–1999, nevertheless, its use has been relatively steady since 2005–2009, reflecting its stable use in modelling complex systems like ACC. Similarly, the use of other OR areas has stayed relatively stable from 2005 to 2009. Figure 12 portrays a methodological shift over time from stochastic modelling to graph and network methods.

Table 6 presents the number of publications employing each OR area to model each domain. The application of stochastic modelling methods within healthcare settings is noteworthy, reflecting the inherent uncertainty of healthcare processes. For example, Julien et al. (2020) and Wu et al. (2023) utilise Markov models to forecast the prevalence and mortality associated with alcoholic liver disease in the United States and China, respectively. Similarly, Barbosa et al. (2010) implement a Markov model to estimate gender-, age- and consumption-specific mortality and morbidity rates for alcohol-related diseases, as well as the corresponding QALYs and lifetime costs. In contrast, graphs and networks are predominantly applied in other domains, particularly in papers focused on modelling behaviours within social networks (French et al., 2018; Lorant & Nicaise, 2014; Marshall et al., 2019; Meisel et al., 2015; Phua, 2011) or exploring traits and characteristics associated with different alcohol consumption behaviours (Gauld et al., 2023; Ingram & Finn, 2022; Taylor et al., 2021).

**Table 6.** Number of publications utilising each OR area by modelling domain.

OR Method	Domain				Total
	Healthcare	Social Care	Crime	Other	
Graphs and Networks	12	7	0	53	72
Simulation	12	6	18	29	65
Stochastic modelling	21	1	1	18	41
Other	16	5	1	14	36
Total	61	19	20	114	214

**Figure 13.**
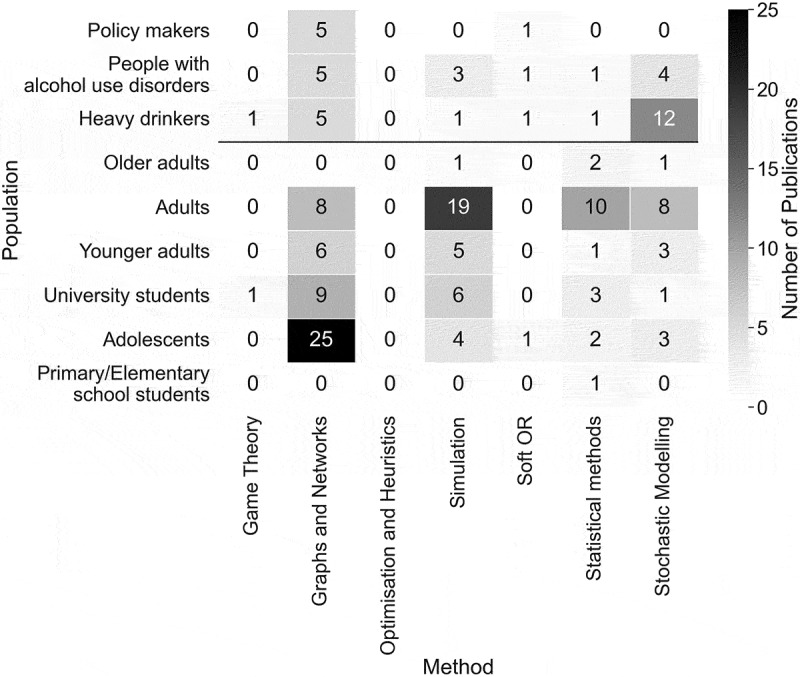
Heat map of the number of publications utilising each OR area to model each population.

Figure 13 is a heat map of the primary OR area utilised and population modelled, remembering that more than one population could be modelled in a paper. The use of graph and network methods to model adolescents’ alcohol drinking behaviours is prominent, conveying the critical influence of social and peer networks during adolescence. Simulation methods are most often used to model a general adult population. For example, Gibbs et al. (2022) and Summan et al. (2020) use simulation methods to test population wide economic alcohol policies. Statistical methods are also used often to model adult populations. Stochastic modelling, the third most commonly utilised OR area, is frequently used to model heavy drinkers, including Laramée et al. (2016) who model the cost-effectiveness of incorporating Nalmafene, medication to support addictive behaviours, into the treatment of alcohol dependence.

Secondary data is the most common source of information used in the literature, and this data source has been used fairly consistently across the most commonly utilised OR areas, portrayed in Table 7. Primary data sources are recurrently used to populate graph and network models, and many of these publications are based on school populations (Huang et al., 2014; Knecht et al., 2011; Marshall et al., 2019; Mathys et al., 2013). Expert opinion has typically been employed to parameterise simulation models.

**Table 7.** Number of publications utilising each OR area and data/information source.

Method	Data/Information Source			Total
	Primary	Secondary	Expert Opinion	
Graphs and Networks	26	38	0	64
Simulation	5	37	8	50
Stochastic Modelling	6	30	2	38
Statistical methods	3	19	1	23
Soft OR	0	4	4	8
Game Theory	1	1	0	2
Optimisation and Heuristics	0	1	0	1
Total	41	130	15	186

### 4.6. Alcohol problem modelled

In this section, we look at the alcohol problem modelled and how this compares to other categories of the taxonomy.

**Figure 14.**
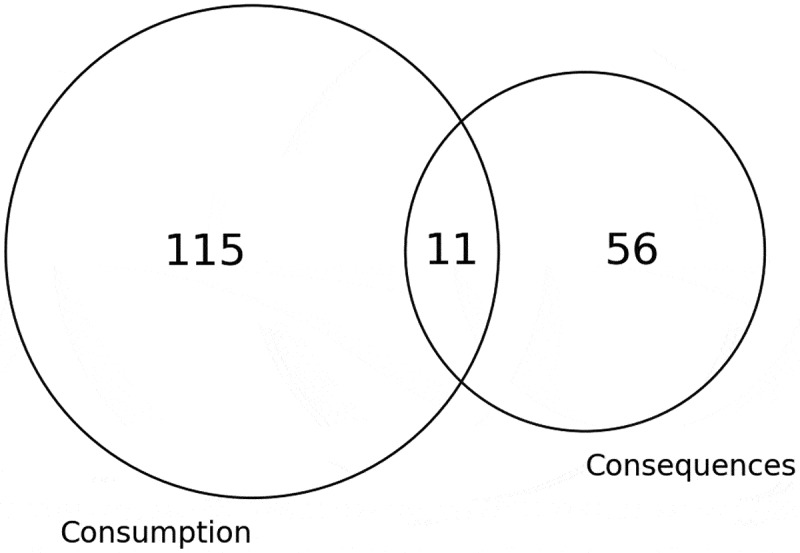
Venn diagram of the problem modelled.

The alcohol problem modelled can be broadly categorised as alcohol consumption or the consequences of drinking alcohol. Most papers focus on modelling consumption behaviours, whilst less publications centralise their research around the consequences and harms of alcohol consumption (Figure 14). Furthermore, much fewer publications consider both the consumption and consequences, including examining how certain consumption behaviours may result in specific harms, and how additional factors may influence the way in which alcohol consumption affects the possibility of experiencing alcohol-related harms. The literature that models consumption behaviours examines drinking trajectories (Fortin et al., 2016; Witkiewitz et al., 2010, 2014) and peer and network influences on alcohol consumption (Azizi et al., 2021; Lorant & Nicaise, 2014; Ragan et al., 2014), whereas the consequences modelled include drink driving (de Carvalho Ponce et al., 2018; Holder, 1998; Salmon et al., 2020; Summers & Harris, 1979), alcohol-related violence (Cerdá et al., 2022; Katerndahl et al., 2021, 2022), and alcohol-related diseases (Gmel et al., 2011; Julien et al., 2024; McCambridge & Golder, 2024; Mendonc¸a et al., 2020). Those papers that considered both the consumption and consequences of alcohol frequently model the effects of a policy intended to alter alcohol consumption and, as a result of this change in consumption, how the policy would affect the consequences (Byrnes et al., 2010; Castillo-Carniglia et al., 2019; Harold, 1998; Keyes et al., 2019; Stacey et al., 2018; Summan et al., 2020; Tawileh et al., 2008).

**Figure 15.**
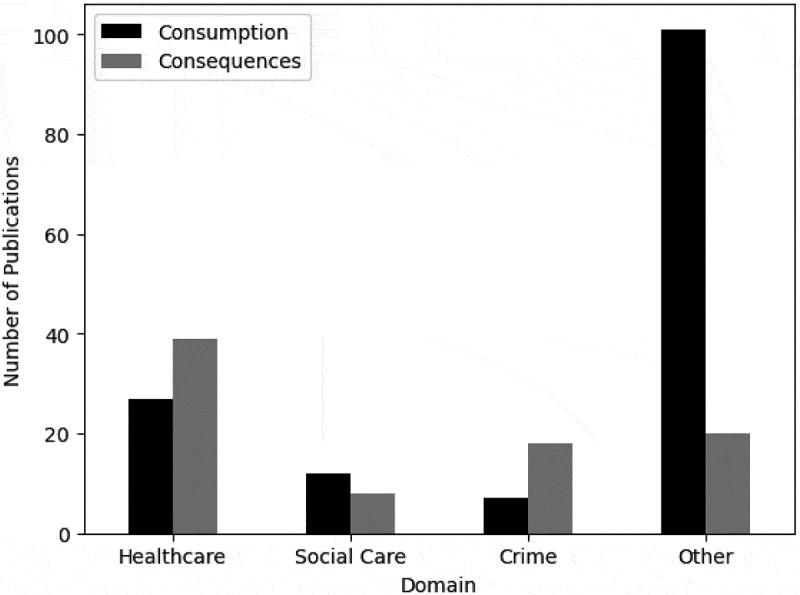
Bar chart of the domain and problem modelled.

We can explore the extent to which each alcohol-related problem has been modelled within each domain, as displayed in Figure 15. Publications that model healthcare and crime domains consider the consequences of alcohol drinking more often than the consumption of alcohol. It must be noted that consumption behaviours have more often been considered in social care settings, nevertheless, not many papers have considered social care domains. The “other” category, consisting of many papers conducting explorations in everyday social settings, model the consumption of alcohol more frequently than the consequences, commonly examining alcohol consumption behaviours in networks (Cintrón-Arias et al., 2009; Hoeben et al., 2021; Poon et al., 2022).

**Figure 16.**
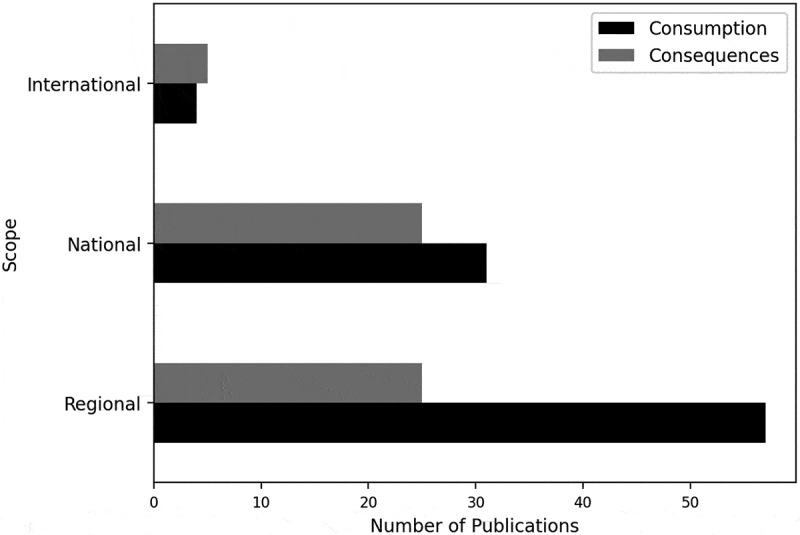
Bar chart of the modelling scope and problem.

When separating the modelling scope by the alcohol problem studied, the greatest difference is observed for regional publications, portrayed in Figure 16. A notably greater number of papers from a regional scope consider consumption. A smaller difference is observed for publications from a national scope, although more of these papers also consider alcohol consumption. The consequences of alcohol drinking have equally been modelled at the regional and national scope, for example, Atkinson, et al. (2018) model the consequences of a range of alcohol-trading policies on acute alcohol-related harms in one state in Australia, whilst Stacey et al. (2018) simulate the impact of an increased taxation policy on health-related consequences in South Africa. For the small number of papers from an international scope, more papers consider the consequences of drinking alcohol compared to the consumption.

Table 8 portrays the number of publications utilising each OR area to model each problem. When considering the most commonly used OR areas, graphs and networks, simulation, and stochastic modelling, the greatest difference in the alcohol problem modelled is observed for graph and network methods, where most papers model consumption. Notably, most OR areas, excluding game theory, are more frequently used to model consumption than consequences. However, it is important to recall that a substantially greater amount of the literature models the consumption of alcohol in comparison to the consequences, as portrayed in Figure 14.

**Table 8.** Number of publications using each OR area to model each problem.

Method	Problem		Total
	Consumption	Consequences	
Graphs and Networks	56	13	69
Simulation	31	25	56
Stochastic Modelling	21	16	37
Statistical methods	14	9	23
Soft OR	3	1	4
Game Theory	0	3	3
Optimisation and Heuristics	1	0	1
Total	126	67	193

## 5. Discussion

In this paper, we developed a taxonomy, searched for and classified literature utilising OR modelling for ACC. Countless consumption patterns and consequences of drinking alcohol are discussed in the literature, therefore we decided to use the acronym ACC to encompass all of these. The taxonomy constructed is primarily based on previous literature reviews in the application of OR methods to model various healthcare settings, whilst being tailored to this paper’s objectives. The taxonomy enabled the literature applicable for full-text analysis to be classified based on categories including descriptive statistics, modelling and methodology, and the alcohol problem modelled.

The search string assembled consists of OR methods, alcohol drinking behaviours and related harms, and relevant JCR categories, and was used to search the Scopus database for relevant literature. Although the search string constructed is extensive, it is not exhaustive, and more alcohol-related harms could be incorporated. However, this might make the search process unmanageable. Machine learning methods were not included in the search because this would have made the search broader than could realistically be conducted, therefore future work could review the application of machine learning methods to model ACC. The search process comprised a clear inclusion criterion, outlined in Section 3. However, any ambiguity regarding a paper’s relevance was discussed amongst all authors to reduce bias. Our search methodology deviates slightly from the PRISMA guidelines. Forward and backward searches were conducted to diversify the search, and these steps were outlined in the flow diagram in Figure 2 to clearly explain how literature was retrieved via other methods. An additional arrow was added between the documents included in the full-text analysis from the forward search and removing duplicates from the backward search to clearly illustrate that duplicates were removed from both the original and forward searches. Moreover, the “removing duplicates” step was removed from the original search as this was redundant. All documents were retrieved and screened therefore this step was not included in the flow diagram. Furthermore, the inclusion criteria were explained in Section 3 hence this was not duplicated in the flow diagram.

### 5.1. Descriptive statistics

The number of publications in this domain has gradually increased since 2005–2009, portrayed in Figure 3, a trend consistent with previous literature reviews in the application of OR methods to model healthcare settings (Howells et al., 2023). Increasing pressures on public services due to an increasing eldery population and inflation may also have encouraged an increase in the amount of research into ACC. We did not include any analysis concerning the continent of the literature because of insufficient information regarding the relationship between the volume of research conducted and the extent of ACC issues. Figures 4 and 5 display the array of research disciplines associated with this research area. The forward and backward searches illustrated the vastness of the research domain, despite this broad range of JCR categories not being incorporated into the search string. Whilst additional JCR categories could have been included in the search string, it is difficult to conclude which categories would be most relevant as there was often only one or two publications in each category. Nevertheless, it is apparent that the research domain is led by the social sciences and public health fields, and less so by the OR field. We can infer that ACC are multidisciplinary issues encompassing multiple research fields, reinforcing the need for interdisciplinary collaboration and a whole systems perspective to effectively model ACC.

Although adults were the most commonly modelled population, adolescents were also frequently considered, illustrated in Figure 6. Adolescence is hypothesised to be a critical period in forming beliefs and behaviours which may relate to alcohol behaviours, justifying the recurrent modelling of this population (Lee et al., 2023). Nevertheless, in 2019, adults aged 25–49 were the age category most at risk to experience the burden of disease from alcohol (GBD 2019 Risk Factors Collaborators, 2020), inferring an over-consideration of adolescents’ drinking behaviours. Alternatively, future research could explore the relationship between adolescent alcohol consumption and alcohol-related harms at older ages.

### 5.2. Modelling domain

As anticipated, not many papers considered healthcare, social care, and crime domains in combination when modelling ACC. In fact, only one relevant publication had done so as portrayed in Figure 7, and this paper was published in 1998 (Holder, 1998). This may be a result of the complexity of the whole system. Although healthcare was the most commonly modelled domain, the specific care area was not often identified, impacting our understanding of the demand of ACC on specific healthcare services. Considerably less papers considered crime and social care settings compared to healthcare settings. Defining social care is difficult and there is no universal definition, which may have affected our domain categorisation. Social care is individually tailored to each country or region’s needs. If a comprehensive definition of social care was established, it is unlikely to remain applicable for long as social care needs continuously evolve to react to society’s ever changing needs (Dickens, 2012; Rode, 2017). The way in which OR researchers collaborated with teams within the domains could be added into the taxonomy to identify the extent of interdisciplinary collaboration, and to highlight the value of integrating OR and public services. This may also help address the lack of implementation.

The most recurrent functional area was relationship influences or modelling, signifying an emphasis on the role of relationships and peer/familial networks. There was scarce use of the functional areas of workforce/staff management, planning and system/resource utilisation, and quality management and performance monitoring or review, which is surprising as these are typical areas of application of OR methods. Modelling these functional areas could be key opportunities to explore the demand of ACC on healthcare, social care, and crime services.

### 5.3. Implementation level

As is too commonly observed, the majority of papers were theoretical, with less being conceptualised, and the research findings scarcely being implemented, displayed in Figure 9, a finding congruous with previous literature reviews in the application of OR methods to model healthcare settings (Brailsford et al., 2009; Howells et al., 2023). Howells et al. (2023) express that indicating the planned implementation of research could encourage the utilisation of OR methods to model healthcare areas. Moreover, greater interdisciplinary collaboration between academics and practitioners could encourage greater implementation of findings. Categorising the implementation level proved difficult, therefore the classification could be simplified into a two step process: firstly, whether the method/model has been discussed with a client or organisation, and secondly, whether the method/results have been used in practice. Alternatively, the labels “not mentioned”, “currently being evaluated”, and “implemented” could be used as suggested by Gjerloev et al. (2024).

ACC presents unique challenges compared to other public health issues due to its common societal and cultural acceptance. Alcohol consumption remains widely promoted and normalised across numerous cultures and countries (Fitzpatrick et al., 2016; McCambridge & Morris, 2019; Probst et al., 2020; Russell et al., 2020). Furthermore, the alcohol industry, with a strong commercial interest, is likely to resist interventions directed at reducing or limiting alcohol consumption, given the likely impacts on their profits (Tawileh et al., 2008). This highlights the need for researchers and practitioners to incorporate considerations of both societal norms and industry opposition when building OR models and exploring interventions, emphasising the need for greater interdisciplinary collaboration to develop a comprehensive understanding of the complex feedback loops within ACC to manage the complexity of implementing effective interventions.

### 5.4. Planning decision level

The planning decision level was irrelevant to most publications, therefore only 31 publications were classified in this category. This small number of publications was also found in a previous literature review in a healthcare setting (Howells et al., 2023). Most publications addressed strategic planning decisions, which is reasonable as many intervention and prevention studies relevant for this paper sought to test and outline long-term policies. Nevertheless, it might be beneficial to examine how greater consideration of all planning decision levels may contribute to a more holistic view of ACC.

### 5.5. Modelling and methodology

With regard to the modelling scope, the majority of studies modelled from a regional scope, displayed in Table 3, reinforcing social networks’ influence on ACC as networks are often regionally established. Additionally, consumption was most often modelled at a regional scope, portrayed in Figure 16, further reinforcing this postulation. Table 3, displaying the number of publications by modelling scope and domain, indicates that healthcare, social care, and crime domains have not consistently been modelled at the same scope – healthcare was most often modelled nationally, whereas social care and crime were most commonly modelled regionally, inferring a relationship between modelling scope and domain.

An abundance of publications were modelled from a societal perspective, with substantially less papers modelling from the patient or provider perspectives. Modelling from the provider perspective, constituting the organisations providing services could provide greater opportunities to explore the demand of ACC on healthcare, social care and crime.

The publications’ outcomes were classified as health, cost, time, and/or behaviours. Behaviours was the main outcome of the majority of publications, suggesting that the literature is primarily directed towards modelling consumption, a theme transpiring throughout this discussion. Greater modelling of health, cost, and time outcomes could reveal more detailed insights into the demand that healthcare, social care, and crime services endure from ACC. The modelling outcome findings align with the functional area and modelling perspective findings, as the exploration of consumption continuously overshadows the consequences.

The utilisation of graph and network methods to model ACC was substantial in the literature, strengthening earlier suggestions of the influence of social networks in alcohol drinking contexts. This may indicate that social networks are pivotal for ACC, revealing the social nature of ACC. Figure 13 portrays the notable use of graph and network methods to model adolescent populations, implying social networks’ pertinence for adolescents’ consumption. Mathematical programming has scarcely been utilised in the literature, depicted by both the small use of optimisation and heuristic methods illustrated in Table 4, and by only one paper’s aim being optimisation, portrayed in Figure 11. This contrasts with other healthcare areas where OR has been applied, including operating room planning and scheduling and home healthcare, where optimisation and heuristics methods have been the most commonly utilised OR area, shown in Table 1. This could outline a gap in the literature in utilising optimisation methods to improve intervention and policy effectiveness. We explored the percentage of publications each year using each OR area, displayed in Table 4. It is apparent that consideration must be given to the number of publications published each year, portrayed in Figure 3, when discussing this finding to ensure that we are not misled. The percentage of publications utilising graph and network methods increases over time, similarly to the total number of publications, confirming an increased use of graph and network methods over time.

Secondary data sources were the dominant data resource utilised in the literature. However, when the data/information source was compared to the OR area utilised (Table 7), the significant use of primary data sources to populate graph and network models was revealed, along with a considerable utilisation of expert opinion to detail simulation models. Primary data may have been used more often with graph and network methods because of the somewhat ease of collecting network connection data, especially in the nomination of friendship ties from a given school roster (Längler et al., 2019). Furthermore, the substantial use of expert opinion to parameterise simulation models highlights the opportunity of utilising expert opinion, including practitioners or policy makers, to understand complex systems when primary or secondary data is not available.

### 5.6. Alcohol problem modelled

The literature was primarily focused on the consumption compared to the consequences of drinking alcohol, reinforcing previous realisations of substantial research on alcohol consumption in social contexts. Only a small proportion of publications considered both the consumption and consequences of alcohol, implying that the way in which different alcohol consumption behaviours result in different harms has scarcely been regarded. Nevertheless, further exploration into the papers considering both the consumption and consequences of alcohol revealed that the majority looked at changing consumption behaviours to alter the consequences. Consequently, it is possible that more researchers are considering consumption only as they may assume that changing alcohol consumption will automatically address the consequences. Figure 16 strengthens previous implications of the relationship between alcohol consumption and social networks as the analysis emphasised the regional foundations of alcohol consumption. This is further underpinned by Table 8 portraying a considerable use of graphs and networks to model alcohol consumption. Simulation was the most common area to model the consequences of alcohol, possibly indicating the complexity in understanding harms resulting from alcohol consumption, and hence more effort has been taken to model the consumption of alcohol.

### 5.7. Missing papers

It is important to acknowledge that some relevant papers did not emerge in this literature search, including system dynamics, complex systems modelling, and Markov modelling papers (Ip et al., 2012; Lee et al., 2021; McGill et al., 2022; Tawileh et al., 2008). This might be a result of the difficulty to entirely capture the literature utilising OR methods to model ACC due to the broad research areas conducting the studies. The field encompasses numerous research fields, as portrayed in Figure 5, which may have different expertise, methodologies and views, potentially resulting in a disconnected group of researchers. This large and disjointed cohort of researchers may therefore not be citing, or are not sufficiently aware of, each other’s research. Alternatively, since only a small number of publications in the field have been published in OR/MS journals, as shown in Figure 4, researchers might have given greater focus to the application of OR methods to model ACC rather than the development of appropriate or improved OR methods in this field. We cannot discount that our methodology could have played a role because as previously expressed, the search string was not exhaustive.

## 6. Conclusion and future work

The objective of this literature review was to construct a taxonomy, collect publications, and examine the application of OR methods to model ACC, to understand the research field and identify areas for future work. Detailed analyses allowed trends in publications to be identified to understand the focus of OR applications in ACC contexts and identify areas for future work. We aimed to clarify the extent of the use of such methods and establish the degree to which ACC has been modelled from a whole systems perspective. A taxonomy was constructed which will support future practitioners and researchers to classify, explore and understand the research that has been conducted in the field.

With regard to the utilisation of OR in ACC settings, a salient finding revealed by the extensive analysis was that graph and network methods was the predominantly utilised OR area to model ACC, one of the many aspects of the analysis that suggested an emphasis on alcohol consumption in the literature. Nevertheless, graph and network methods were mainly used to model adolescents, therefore, future research could clarify how social networks’ effects differ across the lifespan. The modelling outcome was predominantly behaviours, and was less frequently health, cost or time, reinforcing the notion of a focus on alcohol consumption, and the scarce modelling of the consequences of drinking alcohol.

Another key finding of this literature review was the infrequent modelling from a whole-systems perspective as healthcare, social care and crime domains have scarcely been modelled in conjunction. Until whole systems modelling is undertaken, we might not accurately understand the complexity of the alcohol drinking system. The relevant literature was included under several JCR categories, revealing the broad research areas that have strived to model ACC. However, the research field seems to be disjointed, suggesting that future work requires greater interdisciplinary collaboration to most effectively model this complex system.

The gaps identified in the literature and guidance for future work are as follows:

- Healthcare, social care, and crime domains have scarcely been modelled in combination in this context. Modelling multiple domains and utilising a whole systems perspective could provide a more comprehensive understanding of the demand and harms resulting from ACC. This might require researchers to deviate from the currently favoured OR methods in ACC contexts.
- The research field incorporates various research disciplines, including social sciences, public health and OR, however, this cohort of researchers seems to be disjointed. Greater interdisciplinary collaboration could bring together the expertise of these domains.
- Previous research has tended to focus on the consumption of alcohol, including modelling trajectories and the influence of social networks. There is an opportunity in the literature to offer greater attention to the consequences in addition to the consumption of alcohol, to improve understanding of the demand that ACC place on public services, and the relationship between the consumption and consequences of alcohol.
- Our search string was not exhaustive in terms of the consumption behaviours, harms and JCR categories incorporated. Additional influences on ACC could be considered, for example, the economy and taxation.
- Although this paper focuses on the application of OR to model ACC, the taxonomy constructed could be generalised to provide a framework to classify literature utilising OR methods to model other substances or healthcare areas.

In conclusion, ACC results in numerous harms, placing demand on healthcare, social care and crime systems. Comprehensive modelling of ACC could support public services to ensure that sufficient resources are in place, and that prevention strategies are appropriately implemented. This might become increasingly important in time due to the reported shift in alcohol consumption during the COVID-19 pandemic which could increase future demand resulting from alcohol-related harms. The findings of this literature review provide an overview of the way in which OR methods have been utilised to model ACC, and the gaps identified assist to guide a future research agenda.

## Data Availability

The dataset produced in this article can be accessed at https://doi.org/10.5281/zenodo.14051890.

## Appendix A. Search terms

**Table A1.** Search string terms.

Operational Research		Alcohol drinking behaviours	
Agent based model*	Branch and bound	Acute alcohol intoxication	Alcohol abstain*
Branch and price	Column generation	Alcohol abuse	Alcohol addict*
Constraint program*	Constructive heuristic	Alcohol consum*	Alcohol dependen*
Delphi method	Discrete event simulation	Alcohol detox*	Alcohol disorder
Discrete optimi*	Dynamic program	Alcohol drinking	Alcohol excise
Evolutionary algorithm	Game theory	Alcohol intake	Alcohol intervention
Genetic algorithm	Goal program*	Alcohol intox*	Alcohol law
Heuristic$	Integer program*	Alcohol legislat*	Alcohol misuse
Linear program*	Markov chain	Alcohol polic*	Alcohol prevent*
Markov decision*	Markov model	Alcohol pric*	Alcohol recov*
Mathematical model	Mathematical program*	Alcohol-related disorder$	Alcohol restrict*
Memetic algorithm	Metaheuristic$	Alcohol risk	Alcohol tax*
Mixed integer program*	Monte carlo simulation	Alcohol treat*	Alcohol use
Multi-objective program*	Network analysis	Alcohol use disorder	Alcohol withdraw*
Operational management	Operational research	Alcohol-attributable disease$	Alcoholic
Operations management	Operations research	Alcoholic beverage	Alcoholism
Optimi*	Problem structuring	Alcohol-related crime	Alcohol-related disease$
Quadratic program*	Queueing	Alcohol-related harm	Alcohol-related mortality
Queuing	Robustness analysis	Alcohol-related suicide	Binge drink*
Scatter search	Scheduling	Chronic alcohol intoxication	Chronic hazardous alcohol use
Simulated annealing	Soft operational research	Drink driv*	Drinking behaviour
Soft systems methodology	Stochastic analysis	Harmful drink*	Hazard* drink*
Stochastic model*	Stochastic process*	Social drink*	
Stochastic program*	Strategic choice analysis		
Strategic options development and analysis	System dynamics		
Tabu search			

## Appendix B. JCR categories and journals

**Table B1.** JCR categories included in the search and the journals which the relevant literature belongs to.

JCR Categories	Examples
*Criminology & Penology*	Journal of Interpersonal Violence Criminology Journal of Quantitative Criminology
*Health Care Sciences & Services*	BMC Medical Research Methodology Medical Decision Making Journal of Medical Internet Research Statistical Methods in Medical Research Applied Health Economics and Health Policy The Journal of Behavioral Health Services & Research Journal of Mental Health Administration
*Health Policy & Services*	The Journal of Behavioral Health Services & Research Journal of Mental Health Administration Medical Decision Making Applied Health Economics and Health Policy Health Promotion International
*Industrial Engineering*	Ergonomics Human Factors
*Medical Informatics*	Medical Decision Making Journal of Medical Internet Research Statistical Methods in Medical Research Statistics in Medicine
*Operations Research & Management Sciences*	European Journal of Operational Research Omega
*Public, Environmental & Occupational Health*	BMC Public Health American Journal of Public Health Statistics in Medicine The Journal of Behavioral Health Services & Research American Journal of Public Health
	Journal of Epidemiology and Community Health Journal of Mental Health Administration Injury Epidemiology, American Journal of Community Psychology Prevention Science Health Education & Behavior BMJ Global Health International Journal of Environmental Research and Public Health Annals of Epidemiology American Journal of Epidemiology American Journal of Preventive Medicine Preventive Medicine Reports European Journal of Epidemiology Social Sciences & Medicine The Lancet Public Health Journal of Adolescent Health Traffic Injury Prevention International Journal of Public Health Australian and New Zeland Journal of Public Health
**JCR Categories**	**Examples**
*Social Work*	Family Relations American Journal of Community Psychology
*Substance Abuse*	Addictive Behaviors The American Journal of Drug and Alcohol Abuse Journal of Substance Use International Journal of Mental Health and Addiction Addiction, Drug and Alcohol Dependence Substance Abuse Treatment, Prevention, and Policy Drug and Alcohol Dependence Substance Use & Misuse Journal of Studies on Alcohol and Drugs International Journal of Drug Policy European Addiction Research Psychology of Addictive Behaviors, Alcohol and Alcoholism Journal of Addiction Medicine Alcoholism Clinical and Experimental Research
	Journal of Dual Diagnosis Drug and Alcohol Review, Journal of Studies on Alcohol

## Appendix C. Population labels

- *Primary/Elementary school students*
- *Adolescents*
- *University students*
- *Adults*
- *Younger adults*
- *Older adults*
- *Heavy drinkers*
- *People with alcohol use disorders*
- *Policy makers*

## Appendix D. OR area

**Table D1.** Citations of publications by the OR area and method used.

OR Area	Method	Citations
Graphs and Networks	Network analysis	(Afzali et al., 2017; Anker et al., 2017; Baggio et al., 2018; Balestrieri et al., 2018; Barker et al., 2023; Bernstein et al., 2023; Blondino & Prom-Wormley, 2022; Braun et al., 2006; Cintrón-Arias et al., 2009; Conlin et al., 2022; Cusack et al., 2021; D’Amico et al., 2023; Fergie et al., 2019; French et al., 2018; Fujimoto & Valente, 2012; Gauld et al., 2023; Golder & McCambridge, 2021) (González-Alcaide et al., 2016; Hahm et al., 2012; Hilton et al., 2020; Huth et al., 2022; Ingram & Finn, 2022; Karnick et al., 2022; Kools et al., 2022; Lannoy et al., 2021; C.-T. Lee et al., 2023; Li et al., 2024; Logge et al., 2023; Lorant & Nicaise, 2014; Lorant et al., 2016) (Marshall et al., 2019; McCambridge & Golder, 2024; McGlinchey et al., 2022; Meisel et al., 2015, 2018; Papini et al., 2023; Phua, 2011; Poon et al., 2022; Qeadan et al., 2023; Rosenquist et al., 2010; A. M. Russell et al., 2020; A. M. T. Russell et al., 2023) (Rutten et al., 2021; Shim et al., 2020; Sistad et al., 2023; Taylor et al., 2021)
	Stochastic actor-based/oriented model	(Cheadle et al., 2013; Hallgren et al., 2017, 2021; Hoeben et al., 2021; Huang et al., 2014; Knecht et al., 2011; Light et al., 2013, 2019; Long et al., 2017; Mathys et al., 2013; McCann et al., 2019; McMillan & Schaefer, 2021; Mercken et al., 2012) (Mundt et al., 2012; Osgood et al., 2013; Ragan, 2020; Ragan et al., 2014; Van Ryzin et al., 2016; Schaefer, 2018; Schaefer et al., 2021; Wang et al., 2016)
Simulation	Agent-based model	(Atkinson, Knowles, et al., 2018; Atkinson, Prodan, et al., 2018; Azizi et al., 2021; Buckley et al., 2022; Castillo-Carniglia et al., 2019; Cerdá et al., 2022; B. Fitzpatrick & Martinez, 2012; B. Fitzpatrick et al., 2015; B. G. Fitzpatrick et al., 2016; Garrison & Babcock, 2009; Giabbanelli & Crutzen, 2013; Ip et al., 2012; Katerndahl et al., 2019, 2021, 2022; Keyes et al., 2019) (Lamy et al., 2011, 2011; Purshouse et al., 2014; Redfern et al., 2017; Scott, Hart, et al., 2016; Scott, Livingston, et al., 2016; Stankov et al., 2019; Vu et al., 2023)
	System dynamics	(Apostolopoulos et al., 2018; Clapp et al., 2018; Deutsch et al., 2022, 2023; Holder, 1998; Hosseinichimeh et al., 2022; Ip et al., 2012; Matson et al., 2022; Salmon et al., 2020; Tawileh et al., 2008; Gonzalez Villasanti et al., 2021)
	Microsimulation	(Barbosa et al., 2023; Barbosa, Dowd, et al., 2023; Julien et al., 2022, 2024; Probst et al., 2020, 2023)
	Monte Carlo simulation	(Lai et al., 2018; McEachern et al., 2016)
	Other	(Anker et al., 2017; Stacey et al., 2018; Summan et al., 2020; White et al., 2008)
Stochastic modelling	Markov model	(Barbosa et al., 2010; Bartolomeo et al., 2011; Berman et al., 2019; Bertholet et al., 2010; Book et al., 2005; Brick et al., 2017; Delucchi & Weisner, 2010; Fortin et al., 2016; Hu et al., 1997; Julien et al., 2020; Jyani et al., 2019; Kapoor et al., 2009; Laramee et al., 2014; Laramée et al., 2016) (Maruotti & Rocci, 2012; Mitchell et al., 2008; Muennig et al., 2010; O’Doherty et al., 2016; A. J. Palmer et al., 2000; Prisciandaro, DeSantis, et al., 2012; Prisciandaro et al., 2011; Probst et al., 2015; Puka et al., 2023; Ramalingam et al., 2022; Shao et al., 2023; Shirley et al., 2010) (Sluiter et al., 2018; Soto et al., 2017; Wall & Li, 2009; Wilson et al., 2016; Witkiewitz et al., 2010, 2014; Wong et al., 2013; Wu et al., 2023)
	Markov chain	(Cintrón-Arias et al., 2009; Summers & Harris, 1979)
	Markov process	(Lesko et al., 2023)
Statistical methods		(Barbosa et al., 2019; Bendsten, 2019a, 2019b; Byrnes et al., 2010; Feingold et al., 2019; Gibbs et al., 2022; Gmel et al., 2011; Gustavson et al., 2022; Joneydi et al., 2023; Mubayi et al., 2011; Popova et al., 2019; Probst et al., 2018; Rehm et al., 2014; van den Ende et al., 2024) (Ren et al., 2022; Sánchez et al., 2007; K. D. Shield & Rehm, 2015; K. Shield et al., 2020; Wartberg et al., 2021; Yu et al., 2022; Zhao et al., 2023; Zhu et al., 2017)
Soft OR	Delphi method	(Bekkering et al., 2016; Kingston et al., 2009; Ryan et al., 2011; Schopper et al., 2000)
Game theory		(de Carvalho Ponce et al., 2018; Hopthrow et al., 2007; Mendonc¸a et al., 2020)
Optimisation and Heuristics	Goal program/Multi-objective program	(Korhonen & Soismaam, 1988)
